# Case-based learning in undergraduate orthodontic education: A cross sectional study

**DOI:** 10.12688/mep.20096.1

**Published:** 2024-04-30

**Authors:** Asma Shafique, Asad Ur Rehman, Shazia Ibnerasa, Rebecca Glanville, Kamran Ali

**Affiliations:** 1 Orthodontics, Lahore Medical and Dental College, Lahore, Pakistan; 2 Medicine, University of Plymouth, Plymouth, England, UK; 3 QU Health College of Dental Medicine, Qatar University, Doha, Doha, Qatar

**Keywords:** Case-based learning, Dental education, Orthodontics, Undergraduate

## Abstract

**Introduction:**

Student centric learning approaches have been reported to be effective in introducing higher order cognitive skills required by the health professionals. However, learners’ perceptions must be constructively aligned with new learning interventions to achieve a positive impact on their learning. The aim of this study was to explore the learning experiences of undergraduate dental students with case-based learning in orthodontics.

**Methods:**

A case-based learning model was introduced on orthodontic diagnosis and treatment planning for final year students on a Bachelor of Dentistry programme toward the end of their academic year. A survey was conducted to explore the perceptions and experiences of the participants. The research instrument was based on a previously validated questionnaire and included information on demographics and consisted of 12 items aimed at evaluating the benefits and challenges of cased based learning.

**Results:**

All 67 students in the final-year cohort participated in study, yielding a response rate of 100 percent. Participants across the board perceived CBL to be an effective strategy to learn the subject content and helpful in improving the students’ skills in orthodontic diagnosis, treatment planning and team-working. CBL did not pose any significant challenges or barriers to student learning.

**Discussion:**

Participants reported high acceptance of CBL in orthodontic teaching and learning and a positive impact on their educational experiences. CBL was perceived to be an appropriate strategy to enhance the diagnostic, treatment planning and team-working skills of dental students.

## Introduction

Dental graduates are expected to be competent in orthodontic assessment of patients, recognize the treatment needs of patients, and refer them appropriately for the specialist treatment ^1– 3^. Timely referral to specialist orthodontists is essential to achieve optimal treatment outcomes for patients presenting with malocclusions and abnormalities of dento-facial growth ^4^.

Orthodontic education in undergraduate dental programs is undertaken in multiple settings including didactic teaching in classroom and clinical placements which provide a variable level of exposure to undergraduate students ^5^. The existing models provide limited opportunities for active participation to undergraduate students. Students usually remain passive recipients of information in lectures involving large-groups and do not get adequate opportunities for interaction with the teaching faculty. Similarly, clinical exposure of undergraduate students may also be restricted to passive observation of patient management in specialist settings. Unless students are actively engaged in clinical activities, they may not be able to develop core orthodontic skills such as, history taking, clinical assessments, recognizing the treatment needs of patients, treatment planning and referral to orthodontic specialists ^3^. Several recent studies on final year undergraduate dental students and newly qualified dental graduates have reported lack of preparedness to assess orthodontic needs of patients, reflecting gaps in undergraduate dental education ^6– 9^.

Contemporary healthcare education is underpinned by a student-centered approach and focuses on developing critical thinking, problem solving and clinical decision-making skills in undergraduate students ^10^. The aim is to prepare students for a smooth transition into independent clinical practice beyond the temporal confines of university settings and equip them learner agency lifelong learning skills ^11, 12^.

Strategies aimed at enhancing active student participation, boosting motivation of learners, and encouraging them to become independent and lifelong learners include, but are not limited to flipped classrooms, problem-based learning (PBL), case-based learning (CBL) and peer assisted learning, and reflective learning ^13, 14^. These strategies overcome the pedagogical challenges inherent to the teacher-centred approach by encouraging learners to gain knowledge through active participation and empowering them with higher-order thinking skills ^15, 16^. Such instructional techniques are based on the principles of adult learning ^17^ and are believed to promote synthesis of new knowledge and deeper understanding of concepts by active self-directed learning, collaboration and self-motivation in the adult learners ^18, 19^.

CBL is recognized as a learning process in which the students first gain basic knowledge on the topic, and then engage in a problem-solving activity. This instructional design encourages active involvement of the students in creation of new knowledge by recalling the theoretical knowledge and integrating it with the clinical reasoning to solve the clinical problems presented in a given case ^20^. Both CBL and PBL are inquiry-based learning methods, as they involve a clinical case. Mclean (2016) has compared features of CBL and PBL tools succinctly to establish a deeper understanding. CBL was preferred by the faculty and students in a survey conducted at two academic institutes in the USA to assess the effectiveness of two methods due to flexibility, role of teachers as mediators, reduced workload, and more application in clinical practice ^21^. PBL on the other hand, is a student led learning strategy with students identifying the learning objectives, while the PBL facilitators have a less active role. The benefits of CBL are being recognized increasingly in medical education leading to a more widespread use in undergraduate education ^22^.

### Conceptual framework

The conceptual framework of this study was informed by the lens of constructivist theory. According to the constructivist approach, learning is an active process, and the learner is internally motivated to construct new knowledge and incorporate it in the previous understanding ^23^. Social constructivists like Vygotsky (1978) proposed that “multiple truths are constructed by and between people,” and the truth is “socially and experientially based” ^24, 25^. The learners assume a central role in the field of medical education, as they are responsible for identifying their learning needs. They are motivated to construct new knowledge required to solve the health-related problems in a clinical context through active interaction with other learners. The teacher acts as a facilitator and serves to move students into “areas of proximal development” by providing clinically oriented challenging tasks ^17, 23^. Principles of CBL have been related with the features of constructivist learning environments developed by Cunningham and it was concluded that the learning acquired through CBL intervention aligns well with the constructivist scheme ^26^. Therefore, CBL may be preferably employed for teaching health sciences, including dentistry from a constructivist approach ^21^. It has been established that the learning styles and preferences of the learners must be considered to warrant effective transfer of knowledge with any educational intervention ^27^.

The aim of this study was to evaluate the impact of CBL in orthodontics in an undergraduate dental programme and the objectives were to explore the contextual factors required to implement CBL as an acceptable, effective, and feasible learning strategy in undergraduate orthodontic education.

## Methods

### Ethics

The ethical approval of this study was obtained from the Institutional Review Board (IRB), Lahore Medical and Dental College (Ref. Number FD/84/2023). Written informed consent was obtained from each participant. All data were collected and analysed anonymously.

This study was a cross sectional analytical study; with participants recruited from final year Bachelor of Dental Surgery (BDS) students, from Lahore Medical and Dental College, University of Health Sciences, Lahore, Pakistan. The eligibility criteria were students who were actively pursuing their final year BDS programme. Students who had interrupted their studies were not eligible to participate.

The sample size for this study was determined using a power analysis with G*Power software version 3.1. Using α=0.05, maintain a power of 0.8, and detect small-moderate effects (w=0.3), the minimum sample size required was estimated to be 64 participants.

A probability sampling technique was used to invite undergraduate final year dental students. The study was completed from 5 ^th^ December 2022 to 12 ^th^ February 2023.

A 14-item questionnaire was developed by the research team to evaluate students’ skills in critical thinking, problem solving and clinical decision-making. The first two questions were related to demographics and included information on the gender and educational background of the participants. Questions 3–7 were related to student satisfaction and Questions 8–14 were related to the barriers and challenges experienced by the students during the activity. Responses were recorded using a Likert scale from 2 to -2; strongly agree (2), Agree (1) Neutral (0), disagree (-1) and strongly disagree (-2). Negatively-phrased items (8–14) were reverse scored. The external validity of the questionnaire was established with subject experts to ensure that the items were relevant to the educational goals and objectives in undergraduate orthodontic education. The questionnaire was subsequently piloted to determine the language of the items was clear and comprehensible and the questionnaire length was appropriate. Also, the participants in the pilot were asked to identify any language or grammatical errors and ensure the instructions to the participants were phrased clearly. Appropriate changes were made to the questionnaire based on the feedback by the participants. The final version of the questionnaire was used for data collection.

### Participant activity

The intervention was based on CBL activities in orthodontics. Nine real patient cases were prepared by the research team to encompass the diagnosis and treatment planning of distinct types of malocclusions including class l, class ll, class lll, open bite, crossbite, and deep bite. Each case was accompanied by relevant artefacts including study casts, cephalograms, panoramic radiographs and clinical photographs. Prior to the CBL activity, an orientation session was conducted to brief the participants about the aims, and scope of the study along with information regarding the learning activity. A calibration session was also conducted for the instructors to communicate the objectives of CBL activity, and guidance on facilitating a small group activity. Answer keys for all cases were shared with the instructors to ensure consistency in feedback to the students.

The students were divided into small groups and each group was given one-hour to assess one case for the CBL activity. The tutors distributed the cases and questions to each group. The activity sheets along with answers were returned by the students at the end of the session. This was followed by student presentations and a feedback session which was attended by all students. A senior faculty member moderated the session. A nominated leader from each group presented individual cases along with their responses to questions. At the end of student presentations, the answer keys were shared with the students for self-evaluation and reflection. This was followed by detailed feedback by a senior faculty member. At the end of the session, the participants completed the questionnaire on paper anonymously.

### Data analysis

All data were analysed and visualised using RStudio (version 2022.02.3+492) incorporating R version 4.0.5. Descriptive statistics including 95% confidence intervals were calculated for each item and for the combined dataset. A two-way Analysis of Variance (ANOVA) was used to determine any significant variation between the results by gender and educational background. Estimated marginal means were calculated from the ANOVA outcomes.

## Results

A total of 67 participants were included in the study. Of these, 24 were male (35.82%) and 43 were female (64.18%) yielding a response rate of 100% ^28^. Fifty-two students entered the programme with the Higher Secondary School Certificate (77.61%) and 15 with the Cambridge International Evaluation (22.39%).

The questionnaire comprised fourteen items with question 1 and 2 relating to demographic factors (gender and educational background). Questions 3–7 were positively phrased and so positively scored. Questions 8–14 were negatively phrased and so reverse scored.

The overall mean score for all items was 0.549 (95%CI 0.183-0.914). The mean score for Q3–Q7 was 1.253 (95%CI 1.068-1.439) and the mean score for Q8–Q14 was 0.045 (95%CI -0.249-0.339). Descriptive values for each individual item are summarized in Table 1.

**Table 1.** Descriptives (all respondents).

Item	Mean	StDev	95% CI (lower)	95% CI (upper)
3. This learning activity addressed my learning needs	1.33	0.66	1.17	1.49
4. My instructors communicated the subject content effectively	1.42	0.68	1.25	1.58
5. This learning activity developed my problem-solving skills	1.39	0.74	1.21	1.57
6. This learning activity helped me develop my team-working skills.	1.1	0.84	0.9	1.31
7. This learning activity helped treatment planning skills	1.03	0.82	0.83	1.23
8. I was not adequately prepared for the learning activity	-0.48	0.97	-0.72	-0.24
9. The tools and materials for the learning activity were not appropriate	0.13	1.19	-0.16	0.43
10. I was not sure about the objectives of the session	0.27	1.2	-0.02	0.56
11. I did not receive sufficient feedback during the learning activity	0.45	1.22	0.15	0.75
12. I did not have sufficient time to prepare my case	-0.57	1.13	-0.84	-0.29
13. The questions were not sufficiently clear to me	0.34	1.19	0.05	0.63
14. Artefacts provided for the session were inadequate	0.16	1.15	-0.12	0.44

**Figure 1.**
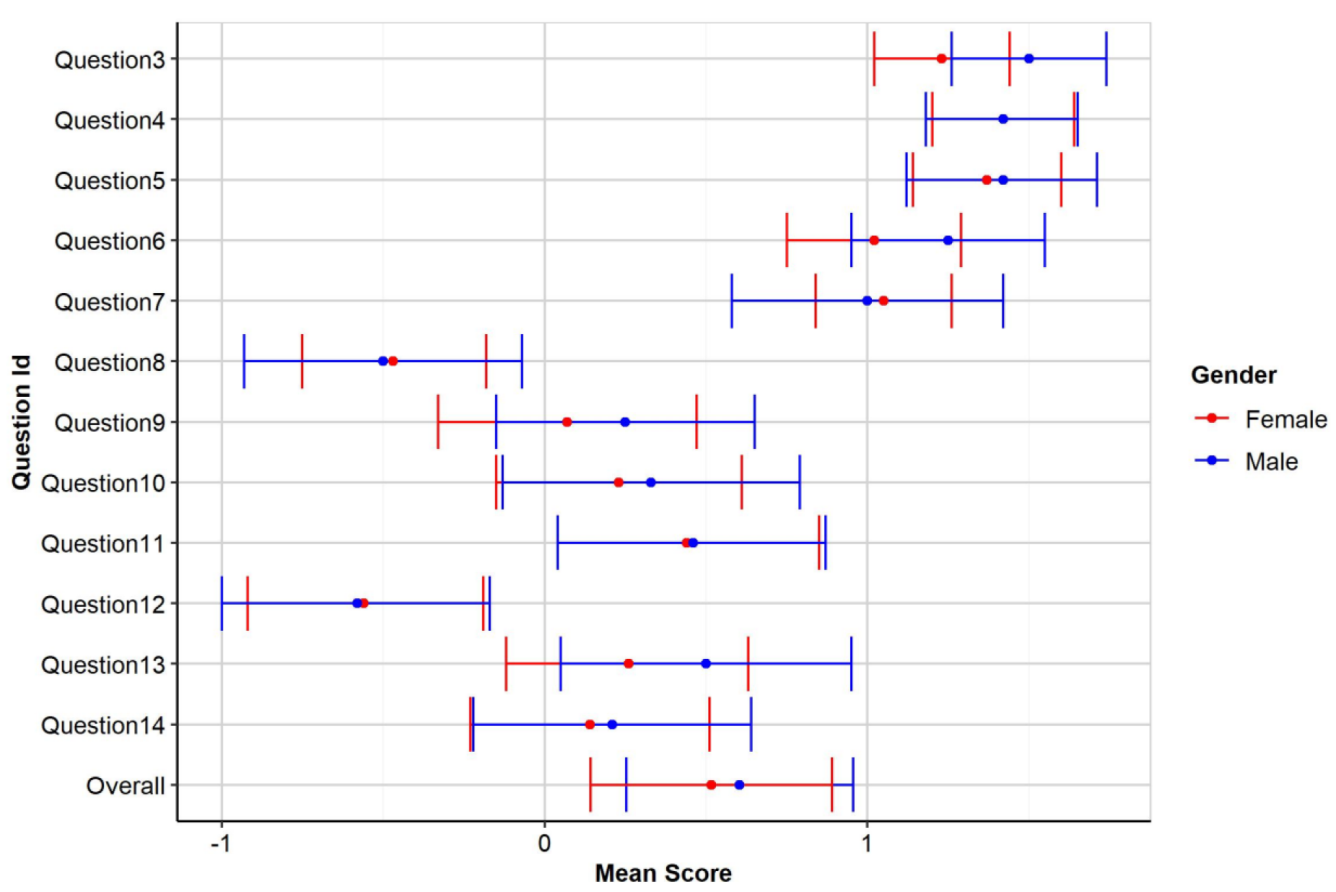
Mean score of participants by gender.

Descriptive values for each individual item, by gender depicted in Figure 1.

**Figure 2.**
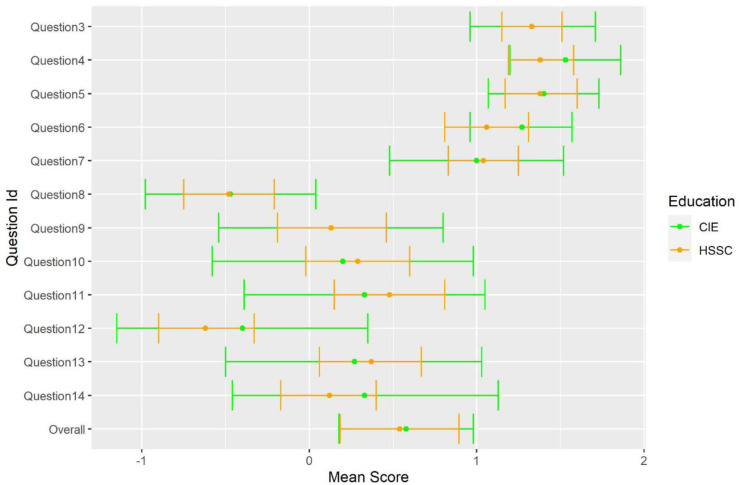
Mean score of participants by educational background.

Descriptive values for each individual item, by educational background are depicted in Figure 2.

Descriptive values for each group of questions (all questions combined, Qs3–Q7 and Qs 8–Q14) can be found in Table 2.

**Table 2.** Descriptives (all items).

	Factor	Mean	StDev	95% CI (lower)	95% CI (upper)
All Questions	All	0.55	1.20	0.18	0.91
	Female	0.51	1.23	0.14	0.89
	Male	0.60	1.15	0.25	0.96
	CIE	0.58	1.32	0.18	0.98
	HSSC	0.54	1.17	0.19	0.90
Q3–7	All	1.25	0.76	1.07	1.44
	Female	1.22	0.76	0.99	1.45
	Male	1.32	0.76	1.09	1.55
	CIE	1.31	0.74	1.08	1.53
	HSSC	1.24	0.77	1.00	1.47
Q8–14	All	0.05	1.21	-0.25	0.34
	Female	0.17	1.25	-0.37	0.40
	Male	0.10	1.12	-0.25	0.44
	CIE	0.06	1.39	-0.37	0.48
	HSSC	0.04	1.15	-0.31	0.39

Analysis of variance identified no significant variation by Gender or Educational Background for all items, items Q3–Q7 or items Q8–Q14 as shown in Table 3.

**Table 3.** ANOVA (all questions).

Factor	Df	Sum of Sq	RSS	AIC	F-statistic	P-value
Gender	1	1.421	1158.910	297.971	0.983	0.322
Education	1	0.229	1157.718	297.144	0.159	0.691

## Discussion

This study explored the impact of CBL on the learning experiences of undergraduate students in orthodontics. CBL has been used widely in medical education and a recent meta-analysis has shown that it is a dynamic instructional approach that is effective in instructing medical students and enhancing their competency in analysing clinical cases ^29^. CBL has also been reported to be an authentic strategy to enhance clinical reasoning skills of medical students ^30^. However, CBL has been used less frequently in Dentistry and this is one of the few studies which explores its application in undergraduate orthodontic education ^31^.

It has been recognized that clinical dentistry requires application of knowledge for clinical problem-solving and a dental graduate is expected to operate at Bloom’s level of *synthesis* ^32^. The results of the current study show that the participants across the board considered CBL to be a suitable tool to enhance their learning experiences. The highest mean score was observed for CBL to be an effective strategy to learn the subject content. The mean scores for items 5,6, and 7 which showed that the participants agreed or strongly agreed that CBL improved their skills in problem-solving, treatment planning and team-working.

Regarding the challenges and barriers related to CBL, the mean scores of the participants to items 8–14 show that CBL did not pose any major challenges for most participants. A vast majority of the participants considered the preparatory information and instructions for CBL to be adequate and the feedback received was also appropriate. Moreover, the educational background of the participants did not show any significant difference in the scores indicating that CBL is an appropriate tool for students from different educational backgrounds in the current cohort of participants.

Our findings corroborate with a previous randomised controlled trial which demonstrated CBL to be more effective in enhancing the orthodontic diagnostic skills of undergraduate students compared traditional lecture-based education studies ^31^. It has also been reported that CBL can enhance critical thinking, motivation, and self-confidence of students ^33^. These benefits of CBL may translate into improved self-efficacy of students which is crucial for lifelong learning. As CBL has been shown to be an effective learning strategy for the construction and synthesis of new knowledge, it may be employed more widely in dental education.

A recent scoping review on undergraduate curricular in orthodontics identified several studies which reported gaps in orthodontic diagnostic and treatment planning skills of dental students as well as inconsistencies in clinical exposure of dental students ^3^.

Given that CBL appears to be an appropriate tool to enhance the diagnostic and treatment planning skills of students, it can be used effectively to discuss common orthodontic problems encountered in clinical practice and provide an appropriate case-mix for undergraduate dental students. Moreover, it can, at least partially, circumvent gaps and inconsistencies in clinical exposure of dental students due to time and resource constraints.

The demographic profile of the participants indicated a high percentage of females (over 64%) which mirrors the high percentage of female dental students in the West and further a feild ^34^. It is particularly encouraging to see the high representation of females in higher education in developing countries like Pakistan as it reflects an increasingly active role of females in healthcare professions. Female education is a fundamental requirement to enhance women empowerment in the society and the high enrolment of females in dentistry is certainly positive. It is envisaged that current generation of female dental students will be able to contribute further to leadership roles in Dentistry in the future and promote equity, diversity and inclusion in the profession ^35^.

The main limitation of this study is that the data has been collected from a single cohort of students from one institution so the findings may not be generalisable. Moreover, the questionnaire was based on closed ended items only and did not allow capturing the views of the participants in more detail. Inclusion of open-ended items and use of qualitative methods would have been helpful to gain a deeper understanding of the perceptions and experiences of the participants. Nevertheless, a wide range of orthodontic patient cases were used in the intervention to cover core skills in diagnosis and treatment planning of commonly encountered clinical cases in orthodontics. In any case, the findings may not be generalizable and further multi-institution studies are recommended to evaluate the impact of CBL in undergraduate orthodontic curricula.

## Conclusion

Participants reported high acceptance of CBL in orthodontic teaching and learning and a positive impact on their educational experiences. CBL was perceived to be an appropriate strategy to enhance the diagnostic, treatment planning and team-working skills of dental students. Moreover, CBL did not pose any significant challenges or barriers to student learning.

## Data Availability

Open Science Framework: Case-based learning in undergraduate orthodontic education https://doi.org/10.17605/OSF.IO/2N9UB ^28^ This project contains the following underlying data: Student Experiences on CBL in Orthodontics.sav Open Science Framework: Case-based learning in undergraduate orthodontic education https://doi.org/10.17605/OSF.IO/2N9UB ^28^ This project contains the following extended data: Appendix Study Questionnaire.pdf Data are available under the terms of the Creative Commons Attribution 4.0 International license (CC-BY 4.0).
