# Supplementary material for: Case-based learning in undergraduate orthodontic education: A cross sectional study
Source: MedEdPublish (2016). 2024 Nov 5;14:29. Originally published 2024 Apr 30. [Version 3] doi: 10.12688/mep.20096.3 (PMC11380727; doi:10.12688/mep.20096.3)
Supplement: Peer review history

# Reviewer report: Reviewer response for version 3

**DOI:** 10.21956/mep.22120.r41024

**Published:** 2025-03-06

**Authors:** Neha Parikh

**Affiliations:** 1 The University of Texas Health Science Center at Houston, Texas, USA

A relevant study gathering perceptions of dental students regarding the integration of CBL in an orthodontics course. 

- **Is the work clearly and accurately presented and does it cite the current literature?**

Clarity of work presented could be improved with some additional proofreading/editing. For example: Introduction\3rd paragraph\last line: "equip them learner agency lifelong learning skills" I believe they are meaning to say, "equip them with lifelong learning skills." Similarly, correct syntax is needed in methods section, 3rd paragraph, last line. 

 Methods section: 5th paragraph: The questionnaire **did not **assess students' skills in critical thinking, problem solving and clinical decision making. Perhaps they are referring to questions assigned after the CBL activity. This should be corrected/clarified.

 Appropriate literature is cited. 

- **Is the study design appropriate and is the work technically sound?**

Authors mention that the research instrument was based on a previously validated questionnaire (no reference is cited). However, per methods, the survey was only partly validated for content/clarity, and pilot tested. In the absence of detailed analysis of individual survey questions (like PCA and CA), they should perhaps state that the questionnaire was evaluated for content and clarity by experts and pilot tested and refrain from stating that a validated questionnaire was used. 

 Qualitative data is integral to any educational survey of this kind and is lacking in this study. This limitation is mentioned in the manuscript. 

 Quantitative data is adequately presented as figures and tables. 

- **Are sufficient details of methods and analysis provided to allow replication by others?**

More details should be added to the second paragraph of participant activity for replication. What was the group size, how long were group presentations, feedback, self-evaluation, reflection and questionnaire submission sessions? Did all these occur on the same day following the 1-hour CBL activity? This should be clarified. 

- **If applicable, is the statistical analysis and its interpretation appropriate?**
**Are the conclusions drawn adequately supported by the results?**

For Qs 8 and 12, the mean score is negative, that means students did not feel adequately prepared for the learning activity and they did not have sufficient time to prepare their case. Authors have not discussed these data at all. Similarly, for Q9 and 14 about the artefacts, tools and resources provided, mean score range overlaps with neutral, and this should also be discussed. Overall, the discussion section needs to improve and elaborated further in the context of data from individual questions and related with existing literature.

Have any limitations of the research been acknowledged?

Yes

Is the work clearly and accurately presented and does it cite the current literature?

Partly

If applicable, is the statistical analysis and its interpretation appropriate?

Partly

Are all the source data underlying the results available to ensure full reproducibility?

Yes

Is the study design appropriate and is the work technically sound?

Partly

Are the conclusions drawn adequately supported by the results?

Partly

Are sufficient details of methods and analysis provided to allow replication by others?

Partly

Reviewer Expertise:

Educational research (student centered active learning), Scientific research (interrelationships between Alzheimer's disease/dementia, oral health, and systemic health)

I confirm that I have read this submission and believe that I have an appropriate level of expertise to confirm that it is of an acceptable scientific standard, however I have significant reservations, as outlined above.

# Reviewer report: Reviewer response for version 3

**DOI:** 10.21956/mep.22120.r40806

**Published:** 2025-01-23

**Authors:** Chaitanya Puranik

**Affiliations:** 1 University of Colorado Anschutz Medical Campus, Colorado, USA

Thank you for providing me the opportunity to review the manuscript. Please see my review below:

**Highlights:**

 1. It is an important topic to investigate

 2. The paper introduction is well written to identify gaps of knowledge

 3. The study undertaken with sound planning

**Caveats:**

 1. The study lacks rigor or clear design. I am unsure if the study was cross sectional or prospective or longitudinal. The way it is defined seems prospective. 

 2. Since there is only one group/cohort the proportion of responses are not provided. The details of each survey question, or case is not provided. I am unsure as to why ANOVA was used and or stratification was done based on education or gender. Does the author feel that there are any differences based on education or gender. If so, logistic regression should have been employed. The ordinal data is presented as mean which is confusing and seems scientifically deficient. No P values are provided to test the aims of the study. 

 3. The authors have not discussed any potential impact of Dunning-Krugger effect or Hawthorne effect as confounder for the study.

Have any limitations of the research been acknowledged?

No

Is the work clearly and accurately presented and does it cite the current literature?

No

If applicable, is the statistical analysis and its interpretation appropriate?

No

Are all the source data underlying the results available to ensure full reproducibility?

Partly

Is the study design appropriate and is the work technically sound?

No

Are the conclusions drawn adequately supported by the results?

No

Are sufficient details of methods and analysis provided to allow replication by others?

No

Reviewer Expertise:

Oral Biology, Fluoride, Dental Materials, Pediatric Dentistry, and Dental Education.

I confirm that I have read this submission and believe that I have an appropriate level of expertise to state that I do not consider it to be of an acceptable scientific standard, for reasons outlined above.

# Reviewer report: Reviewer response for version 3

**DOI:** 10.21956/mep.22120.r40054

**Published:** 2024-12-09

**Authors:** Shoroog Agou

**Affiliations:** 1 King Abdulaziz University, Jeddah, Saudi Arabia

**Comments for Authors**

1. **Novelty:** **Clearly articulate how this study adds to the existing body of knowledge, particularly in light of prior RCTs.**
2. **Distinction between CBL and PBL:** **Provide a clear comparison in the introduction (in relation to the current context) to aid reader understanding.**
3. **Impact Measurement:** **Include or acknowledge the absence of objective measures of knowledge, skills, or behavior and contextual factors.**
4. **Facilitator Roles and Calibration:** **Explain how facilitators were prepared, calibrated, and their specific role in encouraging inquiry-based learning. Clarify how this differs from a standard case presentation.**
5. **Session Design:** **Address the rationale for time allocation, group structures, and how variations between groups were managed.**
6. **Non-presenting Student Role: Clarify how non-presenting students participated and contributed to the sessions.**
** The study contributes to the discussion on Case-Based Learning (CBL) in dental education, but the novelty of the work is unclear given the prior publication of randomized controlled trials (RCTs) on similar topics. The manuscript does not adequately distinguish between CBL and Problem-Based Learning (PBL) in the introduction, which is essential for understanding the methodology. Addressing these issues would clarify the study's unique contribution to the literature.**
** While the aim to explore the impact of CBL and contextual factors is commendable, the study design measures impact solely through student perceptions without assessing actual improvements in knowledge, skills, or behavior. Additionally, the evaluation of contextual factors is not apparent in the methodology.Several methodological elements need clarification:**

- **The role of facilitators in encouraging inquiry-based learning is unclear, as is how this differs from a traditional case presentation.**
- **There is no detailed account of how facilitators were prepared or calibrated across groups, nor how group variations were controlled.**
- **The allocation of time for activities and its rationale are not discussed in detail, leaving ambiguity about whether this design supports equitable learning.**
** The manuscript lacks critical information for replication:**
- **How non-presenting students participated in the sessions.**
- **The standardization of case objectives and instructions for facilitators.**
- **Whether group structures were systematically established.**
- **Providing these details would enhance the study's replicability and methodological rigor.**
** This critique focuses on qualitative and methodological aspects rather than statistical analysis. However, the study’s reliance on perception-based measures limits its ability to offer conclusive insights about the effectiveness of CBL.**
** The underlying data is accessible in a public repository. However, without sufficient methodological detail, reproducibility remains limited despite data availability.**
** The conclusions that CBL positively impacts student learning are based solely on self-reported perceptions. This limitation, along with the lack of exploration of actual learning outcomes, undermines the generalizability and strength of the findings. Additionally, the lack of clarity on contextual factors and facilitator roles detracts from the study's stated objectives.**

Is the work clearly and accurately presented and does it cite the current literature?

Partly

If applicable, is the statistical analysis and its interpretation appropriate?

I cannot comment. A qualified statistician is required.

Are all the source data underlying the results available to ensure full reproducibility?

Yes

Is the study design appropriate and is the work technically sound?

Partly

Are the conclusions drawn adequately supported by the results?

Partly

Are sufficient details of methods and analysis provided to allow replication by others?

Partly

Reviewer Expertise:

NA

I confirm that I have read this submission and believe that I have an appropriate level of expertise to confirm that it is of an acceptable scientific standard, however I have significant reservations, as outlined above.

# Reviewer report: Reviewer response for version 3

**DOI:** 10.21956/mep.22120.r36896

**Published:** 2024-12-02

**Authors:** Avita Rath

**Affiliations:** 1 SEGi University Kota Damansara, Petaling Jaya, Selangor, Malaysia

The article presents an important contribution to the literature on dental education, particularly in exploring innovative teaching strategies like CBL. While the methodology and findings are sound, certain areas require improvement to enhance the manuscript’s rigor and impact.

1. **Clarity and Presentation:**
  - The manuscript is well-written but requires minor corrections (e.g., replace "a feild" with "afield").
  - Abstract: Include specific quantitative results to summarize the findings more effectively.
2. **Integration with Literature:**
  - While the authors cite relevant studies, the discussion lacks depth in integrating findings with prior research, especially regarding the educational background of participants.
3. **Methodological Details:**
  - Clarify the reverse scoring of negatively phrased questionnaire items for better replicability.
  - Future studies should include qualitative components to capture nuanced student perspectives.
4. **Limitations and Generalizability:**
  - The authors should address how cultural and institutional factors might limit the generalizability of the findings.

Have any limitations of the research been acknowledged?

Partly

Is the work clearly and accurately presented and does it cite the current literature?

Partly

If applicable, is the statistical analysis and its interpretation appropriate?

I cannot comment. A qualified statistician is required.

Are all the source data underlying the results available to ensure full reproducibility?

Yes

Is the study design appropriate and is the work technically sound?

Yes

Are the conclusions drawn adequately supported by the results?

Yes

Are sufficient details of methods and analysis provided to allow replication by others?

Partly

Reviewer Expertise:

I am dental educator with background in periodontology and clinical education,

I confirm that I have read this submission and believe that I have an appropriate level of expertise to confirm that it is of an acceptable scientific standard, however I have significant reservations, as outlined above.

# Reviewer report: Reviewer response for version 2

**DOI:** 10.21956/mep.21914.r38643

**Published:** 2024-09-07

**Authors:** Muhammad Haseeb

**Affiliations:** 1 University of Lahore, Lahore, Pakistan

Well conducted study, it provides useful information to the audience, however needs minor revision or clarification in the following aspects: 

**Results Section:**

 Why is it necessary to mention the overall mean? Since the Q3-7 are positively phrased questions and higher mean reflects higher satisfaction of the students and Q8-14 are negatively phrased questions whereby lower mean reflects that students had less challenges during the CBL activity. 

 Overall mean doesn't provide any impactful information. Authors have already mentioned the mean score of two segments of the questions which makes more sense. 

**Discussion:**

 7th paragraph: spelling mistake of "a feild". It should be written like "afield" where ref #34 is cited. 

 Secondly, authors have not discussed about educational background of the students in the discussion section despite being one of the two contextual factors they have looked at. 

**References:** 

 Ref #28 does not have journal name, issue, volume and page number. DOI provided does not open.

Have any limitations of the research been acknowledged?

Yes

Is the work clearly and accurately presented and does it cite the current literature?

Yes

If applicable, is the statistical analysis and its interpretation appropriate?

Yes

Are all the source data underlying the results available to ensure full reproducibility?

Yes

Is the study design appropriate and is the work technically sound?

Yes

Are the conclusions drawn adequately supported by the results?

Yes

Are sufficient details of methods and analysis provided to allow replication by others?

Yes

Reviewer Expertise:

Dentistry, Dental Education

I confirm that I have read this submission and believe that I have an appropriate level of expertise to confirm that it is of an acceptable scientific standard.

# Response

**Published:** 2024-09-07

**Authors:** KAMRAN ALI

**Affiliations:** QU Health College of Dental Medicine, Qatar University, Doha, Doha, Qatar

The authors would like to thank the reviewer for their constructive feedback. Negatively phrased items were reverse scored; hence the mean scores for all items are accurate.

 The educational background of the participants did not contribute to any significant differences in their scores, this is mentioned in the discussion.

 The typographic error in the discussion has been corrected, thank you for highlighting this. Ref 28; The doi and weblink have been cross-checked and are working correctly.

# Reviewer report: Reviewer response for version 2

**DOI:** 10.21956/mep.21914.r37309

**Published:** 2024-06-07

**Authors:** Zainab Mahmood Al-Jammali

**Affiliations:** 1 University of Babylon, Babylon Governorate, Babylon Governorate, Iraq

I have no further comments to make.

 Zainab Mahmood Aljammali

 University of Babylon

Have any limitations of the research been acknowledged?

Partly

Is the work clearly and accurately presented and does it cite the current literature?

Yes

If applicable, is the statistical analysis and its interpretation appropriate?

Yes

Are all the source data underlying the results available to ensure full reproducibility?

Yes

Is the study design appropriate and is the work technically sound?

Yes

Are the conclusions drawn adequately supported by the results?

Yes

Are sufficient details of methods and analysis provided to allow replication by others?

Yes

Reviewer Expertise:

dentistry , prosthetic, implantology, dental material

I confirm that I have read this submission and believe that I have an appropriate level of expertise to confirm that it is of an acceptable scientific standard.

# Reviewer report: Reviewer response for version 1

**DOI:** 10.21956/mep.21519.r36900

**Published:** 2024-05-27

**Authors:** Zainab Mahmood Al-Jammali

**Affiliations:** 1 University of Babylon, Babylon Governorate, Babylon Governorate, Iraq

Kindly do all these suggestions:

**Abstract:**

 Replace the discussion part in the abstract with the conclusion (there should be no discussion in the abstract, remove it kindly and put the conclusion).

**Methods:**

 Have these undergraduate students received a complete curriculum year course and a basic orthodontic knowledge, from terminology

 and etiopathogenesis to the orthodontic treatment of the child before participating in this study? (Mention about this in the Methods).

**Suggestion for further studies:**

 Assessing students' views on the methods of examining the knowledge gained during a university year and quantified their final results.

Have any limitations of the research been acknowledged?

Partly

Is the work clearly and accurately presented and does it cite the current literature?

Yes

If applicable, is the statistical analysis and its interpretation appropriate?

Yes

Are all the source data underlying the results available to ensure full reproducibility?

Yes

Is the study design appropriate and is the work technically sound?

Yes

Are the conclusions drawn adequately supported by the results?

Yes

Are sufficient details of methods and analysis provided to allow replication by others?

Yes

Reviewer Expertise:

dentistry , prosthetic, implantology, dental material

I confirm that I have read this submission and believe that I have an appropriate level of expertise to confirm that it is of an acceptable scientific standard, however I have significant reservations, as outlined above.
